# Supplementary material for: ECNet is an evolutionary context-integrated deep learning framework for protein engineering
Source: Nat Commun. 2021 Sep 30;12:5743. doi: 10.1038/s41467-021-25976-8 (PMC8484459; doi:10.1038/s41467-021-25976-8)
Supplement: Supplementary file 1 — Supplementary Information [file 41467_2021_25976_MOESM1_ESM.pdf]

Supplementary Information

Supplementary Figures

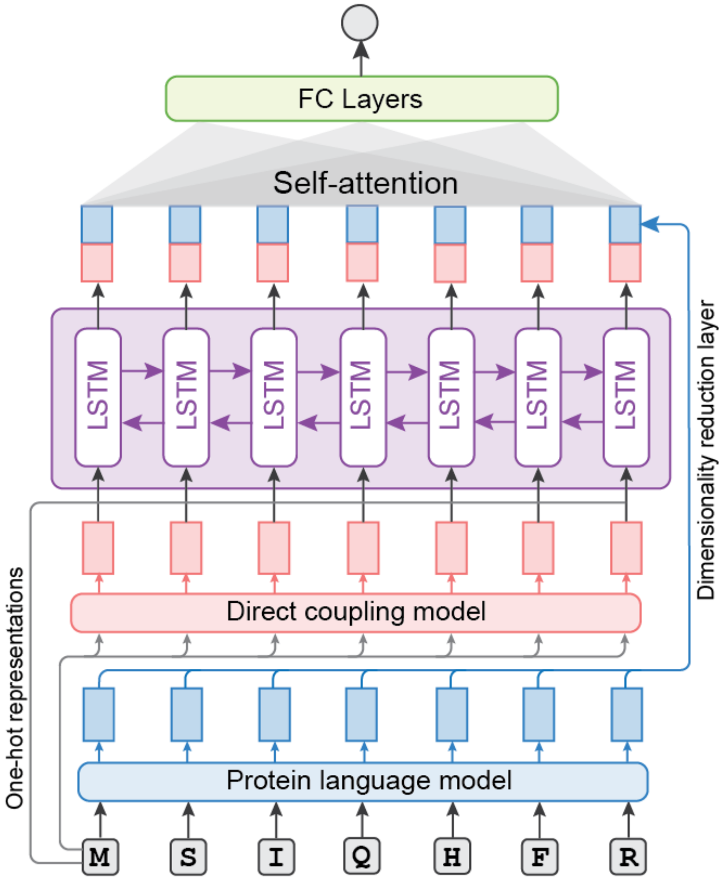

Supplementary Figure 1. The neural network architecture of ECNet. Given the input sequence, the protein language model is used to generate the global evolutionary contexts while the direct coupling model is used to generate the local evolutionary contexts. One-hot representations and the local evolutionary contexts are concatenated and passed to an LSTM. Embeddings produced by the LSTM are concatenated with global evolutionary contexts that have been projected by a linear dimensionality reduction layer. The top layers are composed of a self-attention layer, which summarizes the embeddings of all positions into a single embedding, and fully connected layers that output the final prediction. In the model training, only parameters of LSTM and top layers are updated, while parameters of the protein language model and direct coupling model are fixed. (LSTM: long short-term memory network; FC layers: fully-connected layers.)

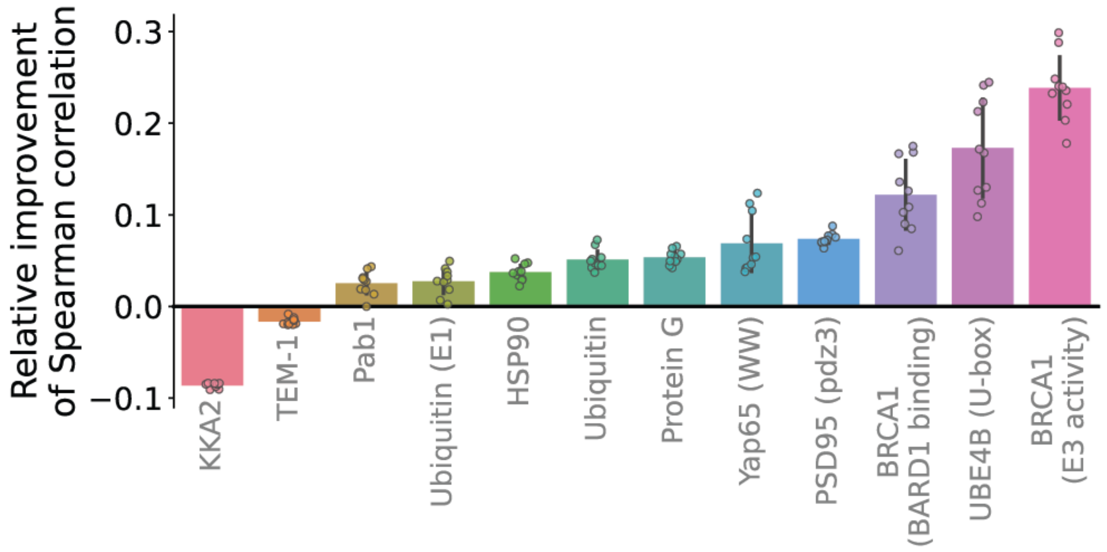

Supplementary Figure 2. Performance improvements on the Envision dataset. This bar plot shows the relative improvements of Spearman correlation achieved by ECNet as compared to the Envision model. Performances were evaluated using ten trials of five-fold cross-validation. The bar plot represented the mean ± SD of the data.

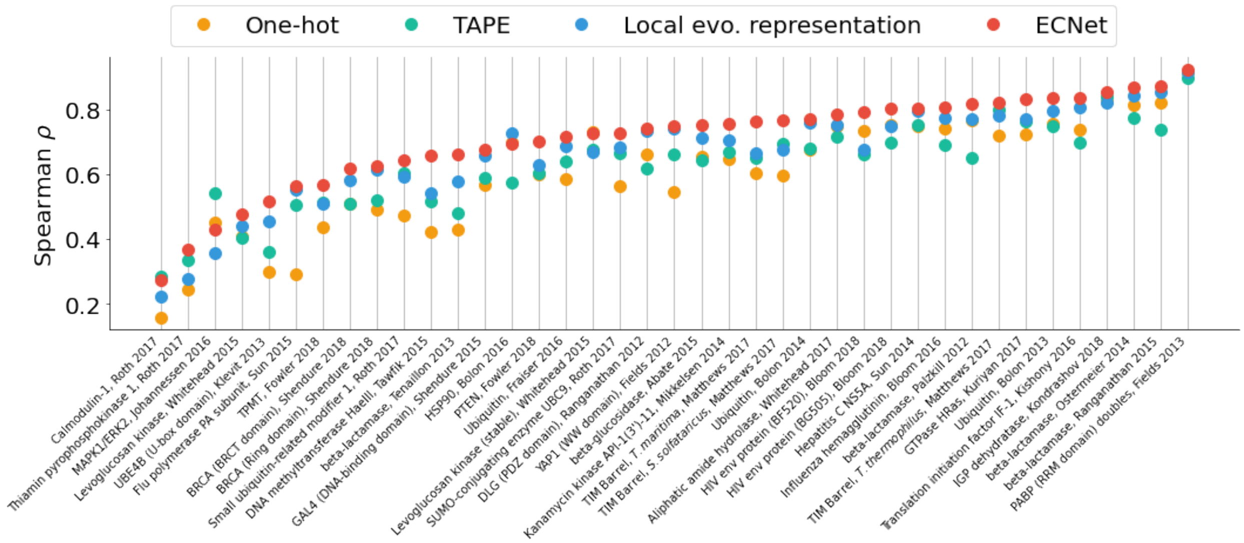

Supplementary Figure 3. Comparison of supervised learning with different representations. ECNet integrated multi-scale sequence representations, including the global evolutionary representation, the local evolutionary representation, and the one-hot representation of a protein sequence. We performed ablation analysis to compare ECNet to models that used individual sequence representation using five-fold cross-validation. Spearman correlation was used as the evaluation metric.

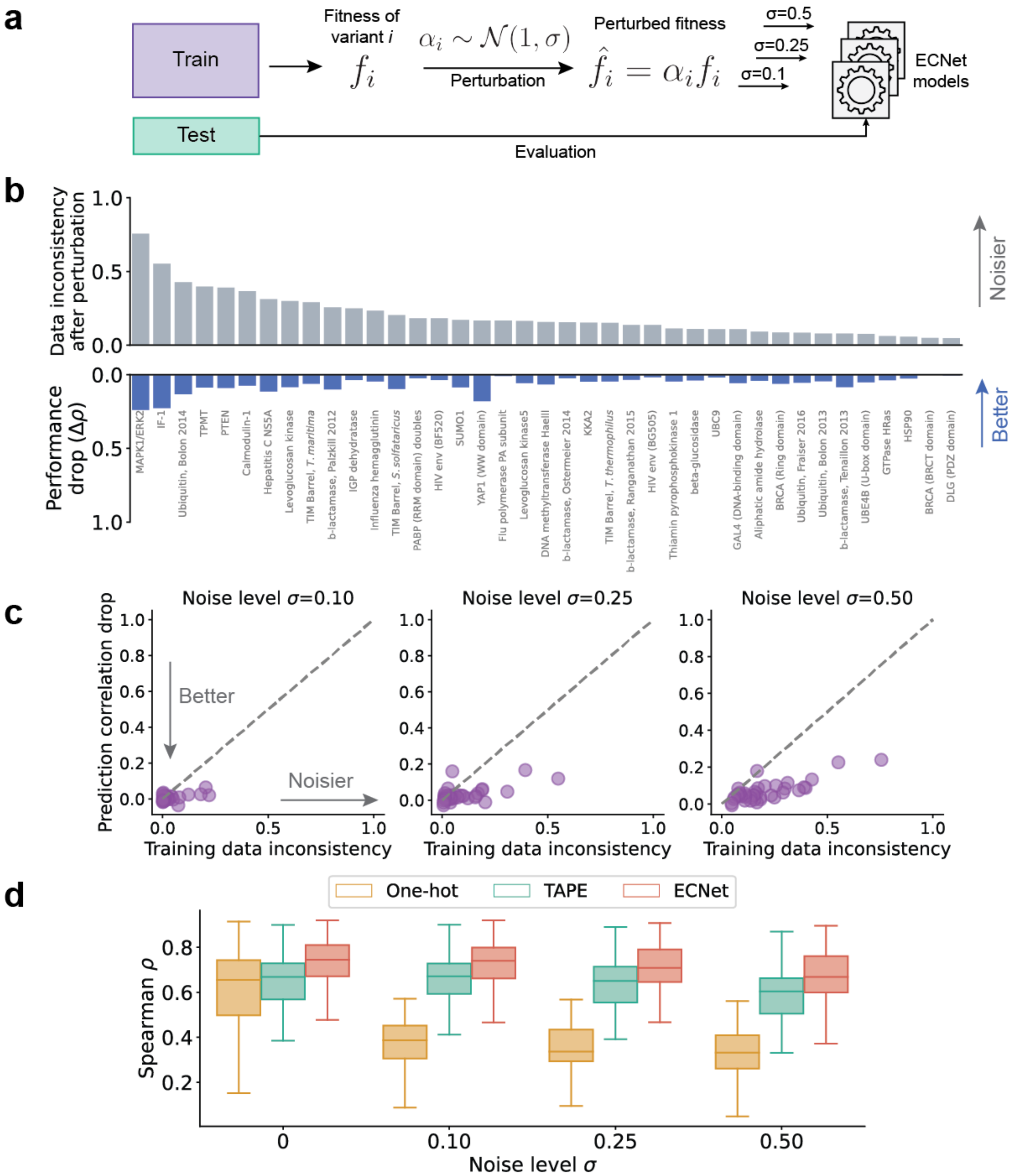

Supplementary Figure 4. Evaluation of ECNet on noisy data. (a) Schematic illustration of the evaluation protocol using simulated noisy data. A random fraction (20%) of the DMS dataset was withheld as the test set and the remaining variants are used as the training set. For each variant in the training set, its fitness value is perturbed by a multiplicative random noise a, i.e., the fitness value f_i of variant i became $\hat{f}_i = \alpha_i f_i$, where α_i was sampled from the normal distribution $N(1, \sigma)$. Three noisy training sets were simulated using $\sigma =$ 0.1, 0.25, and 0.5, and a separate ECNet model was trained on each of the training sets separately. The trained models were then evaluated on the noise-free test set. **(b)** Evaluation results for 39 proteins in the

DeepSequence dataset for $\sigma = 0.5$. Top: data inconsistency caused by the perturbation for each protein. The inconsistency is defined as the $1 - r$, where r is the Spearman correlation between the pre- and post-perturbation fitness values of the training set. A larger inconsistency score means the perturbed fitness data have deviated more from the original fitness values. Bottom: ECNet's performance drop when trained on the noisy training data as compared to when it is trained on the noise-free training data. The performance was evaluated using Spearman correlation. A smaller absolute value ($\Delta\rho$) means the model is more robust to errors in the training data. **(c)** A scatter plot that shows the relationship between the training data inconsistency and the performance drop for every protein in the DeepSequence dataset, at noise levels $\sigma = 0.1, 0.25$, and 0.5. **(d)** A boxplot that compares the prediction performances of ECNet, TAPE, and One-hot models on the DeepSequence dataset at noise levels $\sigma = 0, 0.1, 0.25$, and 0.5. The noise level $\sigma = 0$ means the model is trained on the noise-free training set. Each box plot summarizes the prediction performance on the 39 protein DMS datasets. The midline of box plots represents the median, the lower and upper hinges of the boxes correspond to the 25th and 75th percentiles, and the whiskers extend to 1.5 times the interquartile range from the hinges.

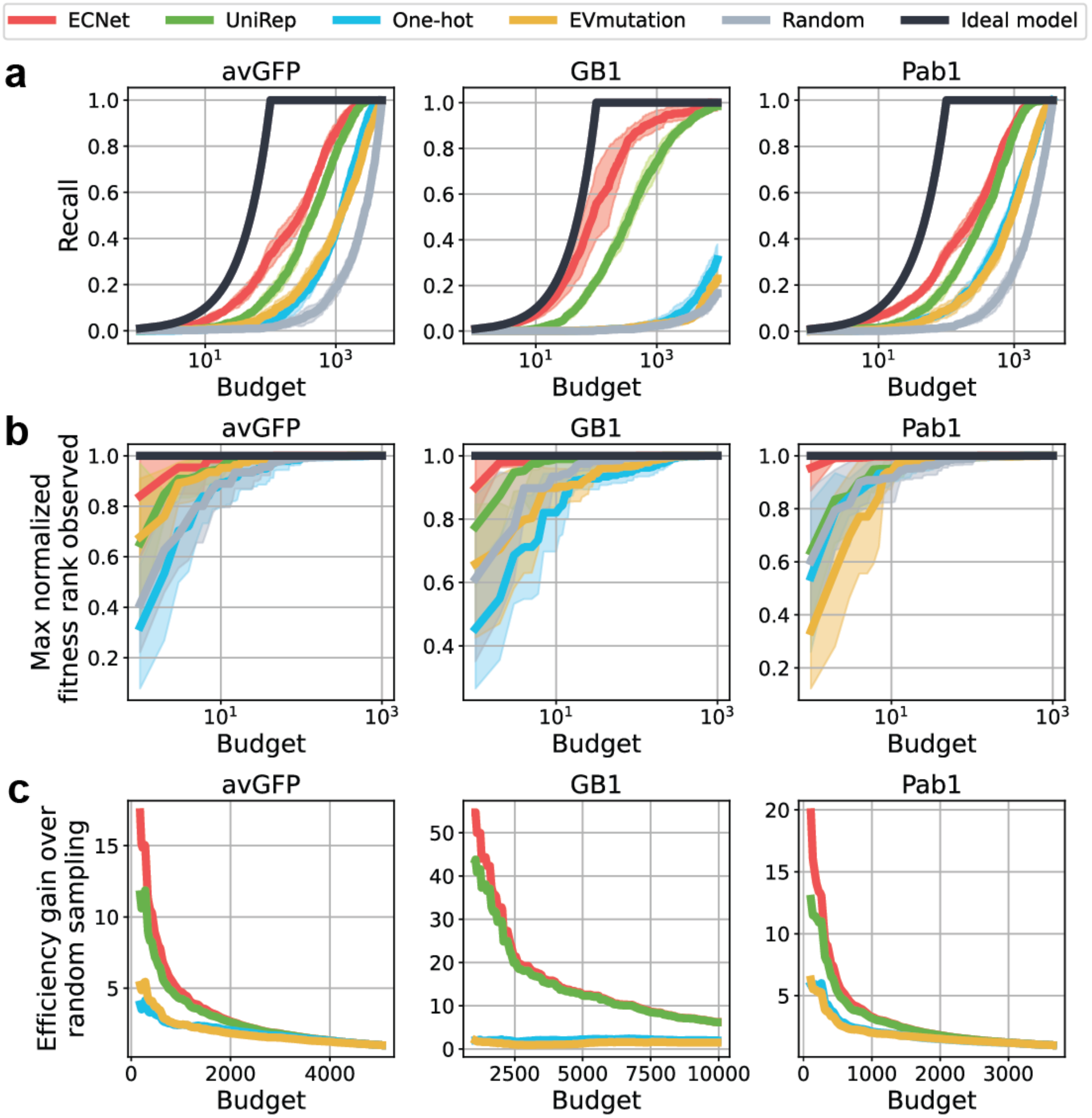

Supplementary Figure 5. Simulation results of using ECNet to prioritize high-performing variants for avGFP, GB1, and Pab1. (a) Recall versus sequence testing budget curves for each method. The recall is defined as the fraction of the true top-100 variants that were ranked within the top *K* predictions of a method, where *K* is a given testing budget (number of variants to test). ECNet was compared to i) UniRep, a supervised method, ii) One-hot, a supervised method that uses simple one-hot sequence representations, iii) EVmutation, an unsupervised method, iv) random model, which is a null model that assigns a random ranking to test variants, and v) ideal model, which ranks the variants using the ground-truth fitness score. **(b)** Maximum normalized fitness rank observed versus sequence testing budget curves for each method. Fitness scores of variants were normalized based on their rank into a value between 0 (the lowest fitness score) and 1 (the highest fitness score). **(c)** Efficiency gain of ECNet, UniRep, One-hot, and EVmutation over the random model with the given testing budget. The efficiency gain is defined as the ratio of a method's recall divided by the recall of the null

model as a function of the testing budget. Error bands in **(a)** and **(b)** depict mean ± SD calculated over 10 independent replicates of the experiments. Curves in **(c)** were smoothed using an averaging window of size 50 along the *x*-axis.

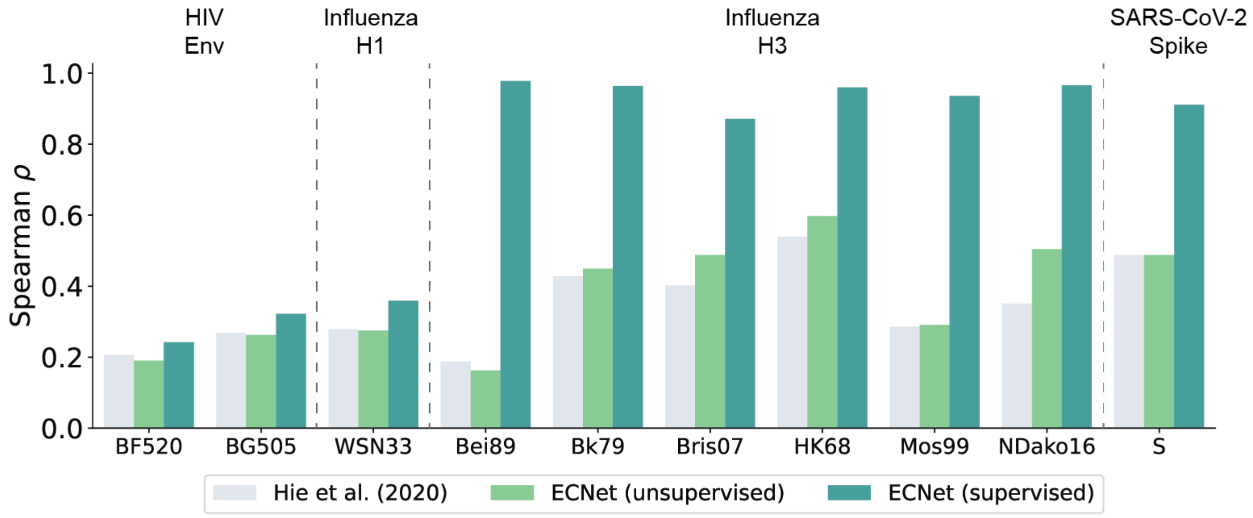

Supplementary Figure 6. Performance of unsupervised and supervised ECNet on viral proteins. The unsupervised ECNet is a model that learns from homologous sequences of the protein of interest to predict how tolerable or favorable a mutation is at a position, and it does not use any fitness data in the training process. The supervised ECNet model predicted the fitness value of the input sequence and was trained using existing fitness data. For reference, the performance of a similar unsupervised model CSCS developed by Hie et al. [25] was also shown. The performance of supervised ECNet was summarized as the average of a five-fold cross-validation.

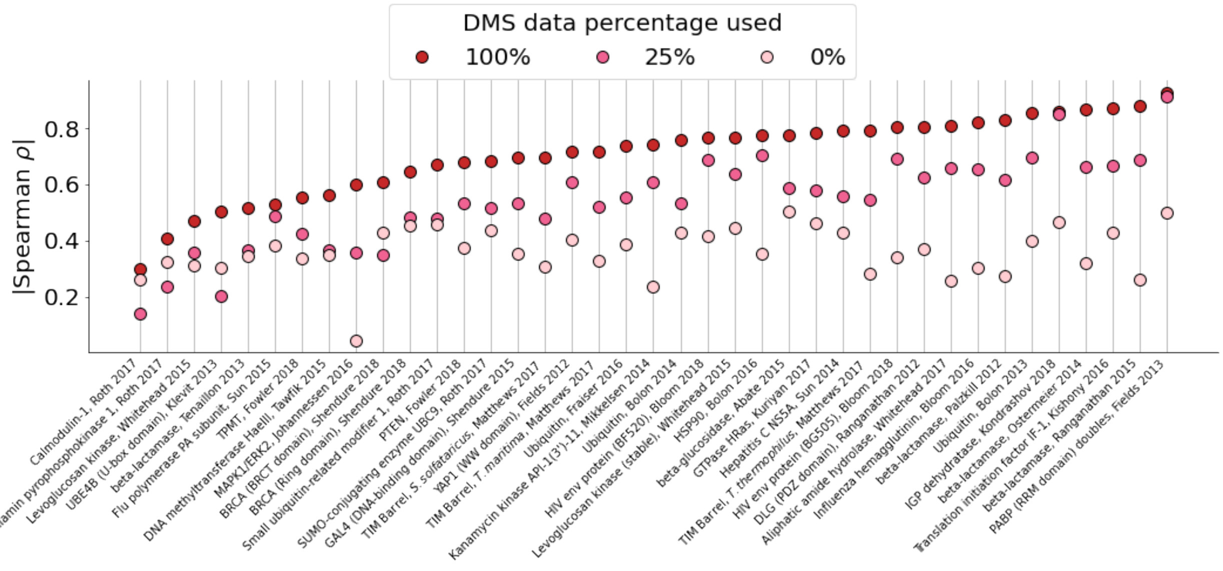

Supplementary Figure 7. Effect of DMS training data size on prediction performance. ECNet was trained in a supervised way with full (100%) or partial (25%) DMS data, or in an unsupervised way (0% DMS data), and then applied to predict the fitness for a holdout set of variants. The supervised ECNet model was trained

using 100% or 25% DMS data, and three model replicas are trained on the same data and their predictions were averaged as the final predictions. The unsupervised ECNet model was trained on homologous sequences of the target protein to predict the probability of an amino acid showing at a position in the sequence (Methods). Performances were evaluated on the proteins in the DeepSequence dataset (Methods).

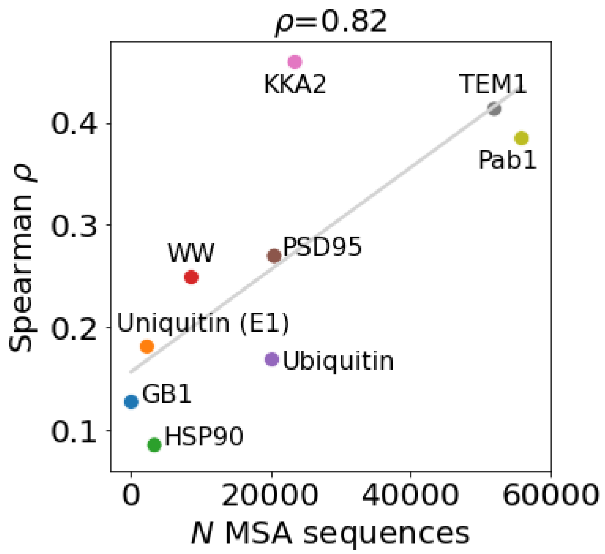

Supplementary Figure 8. Relationships between the number of sequences in MSA and the fitness prediction accuracy using co-variation. Same as in Figure 2b, the changes of co-variation strength (derived from MSA data) were used to predict variant fitness. The prediction accuracy was summarized by Spearman correlation (ρ) for each protein. The prediction performances were then correlated with the number of sequences in the MSA for each protein. Each point in the figure represents one DMS dataset of a protein in the Envision dataset.

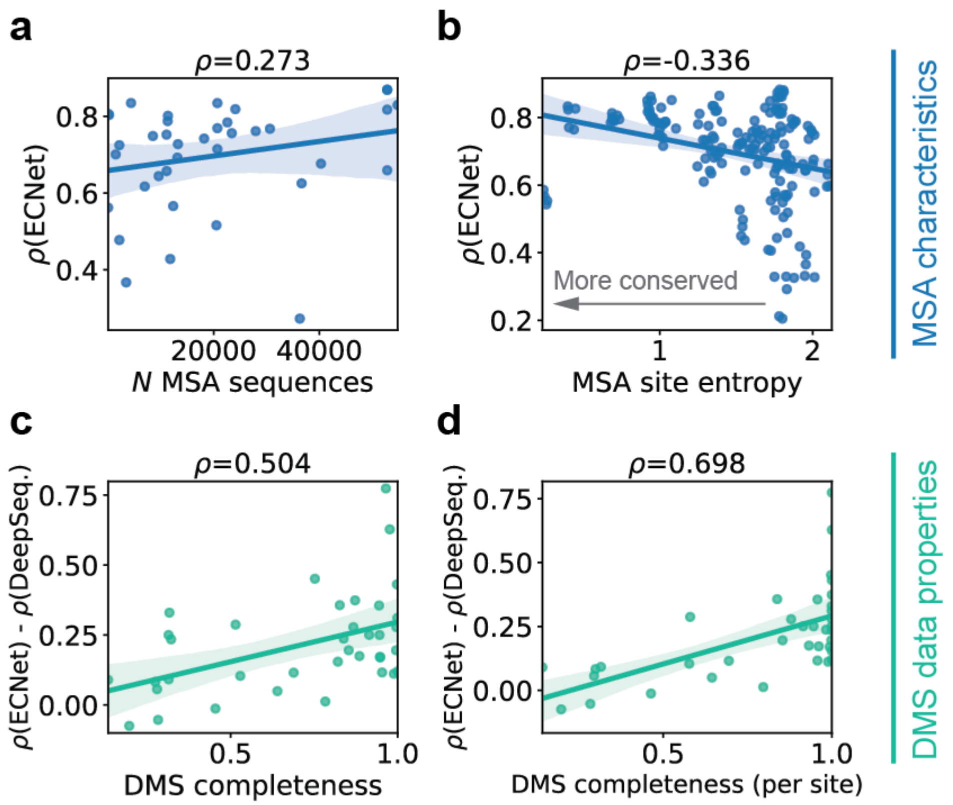

Supplementary Figure 9. Relationships between prediction performance and MSA/DMS data properties. We investigated the relationships between ECNet's prediction performance (evaluated using Spearman correlation ρ) and (**a-b**) MSA characteristics and (**c-d**) DMS data properties. (**a**) Correlation between the number of sequences in the MSA of homologous sequences and ECNet's prediction performance. Each point represents one protein in the DeepSequence dataset. The prediction performance was summarized by the average of five-fold cross-validation. (**b**) Correlation between site entropy in the MSA and ECNet's prediction performance. The site entropy was calculated using the frequency of amino acids at each site in the MSA. Each point represents one fold experiment of the five-fold cross-validation, and its *x*-axis value indicates the average over the entropies of all sites in the test data in this fold. (**c**) Correlation between ECNet's performance improvements against DeepSequence and the completeness of single-mutation protein DMS data in the DeepSequence dataset. The completeness is defined as the percentage of screened variants among all possible single-mutation variants for a protein, i.e., $M/(19{*}L)$, where M is the number of screened variants in the DMS data and L is the length of the protein sequence. Each point represents one protein in the DeepSequence dataset. ECNet's prediction performance was summarized by the average of five-fold cross-validation. (**d**) Same as (**c**) except that the DMS completeness is defined for each site. The per-site completeness is defined as the percentage of screened mutations among all possible single-mutation mutations for a position, i.e., $m/19$, where m is the number of screened mutations for this position in the DMS data. Each point represents one protein in the DeepSequence dataset. The error band indicates the 95% confidence interval of the regression line. The per-site completeness was first averaged over all test sites in a fold and then averaged over all five folds. (MSA: multiple sequence alignment; DMS: deep mutational scanning.)

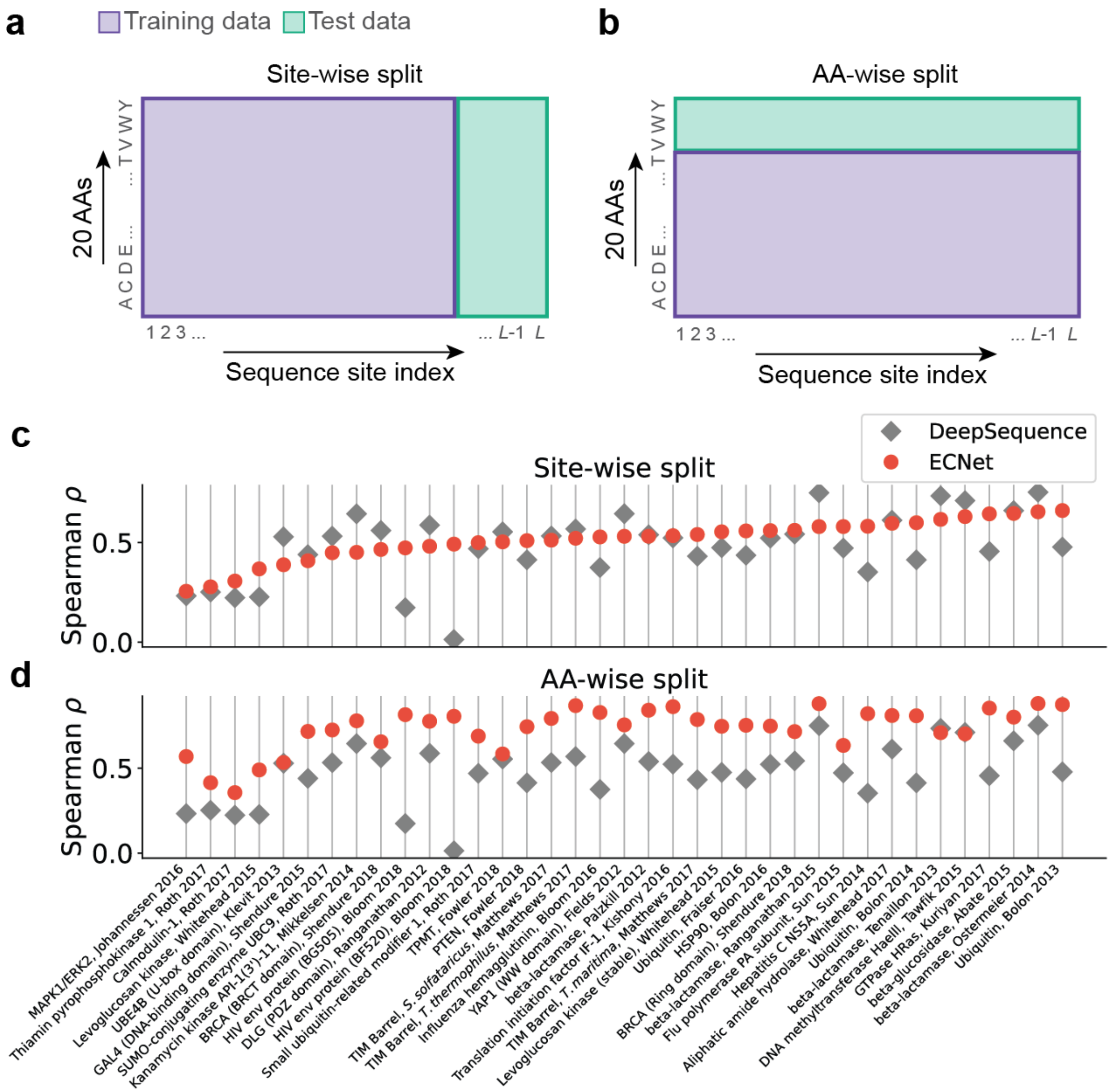

Supplementary Figure 10. Prediction performance on sequence site-wise and amino acid-wise (AA-wise) train/test data split. (**a-b**) Schematic visualizations of train/test split. A DMS dataset is visualized as a matrix where the *x*-axis represents the index of position in the sequence and the *y*-axis represents the amino acid (AA) types the site mutates to. To split strategies are considered: (**a**) Site-wise split: the deep mutational scanning (DMS) dataset was split based on the sequence position (site) in the sequence. Mutants in 80% of the sites were randomly sampled as training data and mutants in the remaining sites were used as test data. The partition of train/test sites was only for the schematic visualization purpose. The actual training sites are randomly sampled and not necessarily the 80% leftmost sites; (**b**) AA-wise split: the DMS dataset was split based on AA types of mutations. For each site, 80% of the mutations were randomly sampled and added to the training set and the remaining 20% mutations were added to the test set. The partition of train/test AA types was only for the schematic visualization purpose. The actual training mutations are randomly sampled and not necessarily the first 80% alphabetically ordered AA types. (**c-d**) Comparison of ECNet to DeepSequence on 37 single-mutation DMS datasets for both (**c**) site-wise split and (**d**) AA-wise split settings. The DeepSequence's

performances are the same in (**c**) and (**d**) as it is an unsupervised model. (AA: amino acid; DMS: deep mutational scanning.)

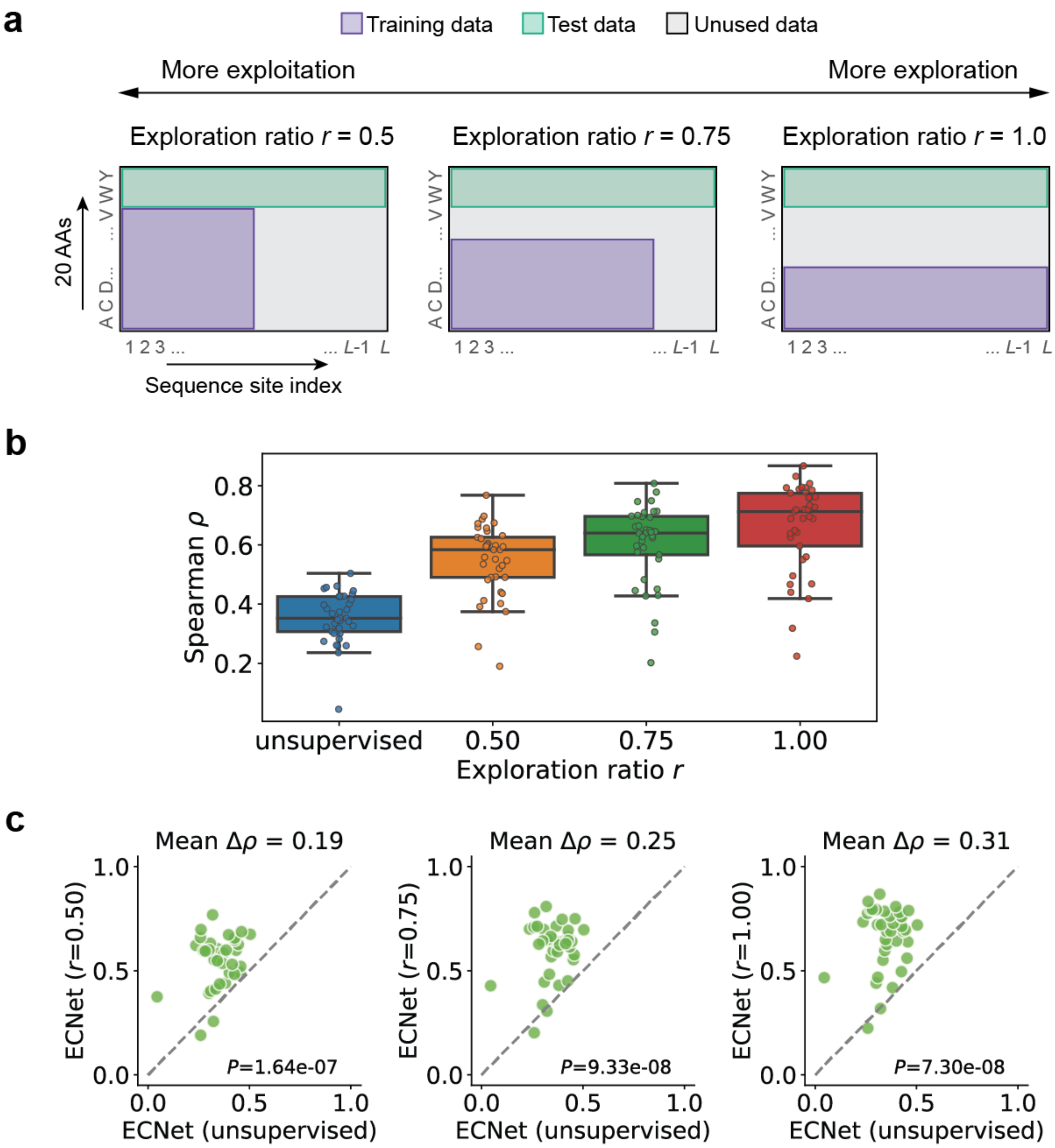

Supplementary Figure 11. The exploration-exploitation trade-off of training data design under a limited experimental budget. Given the limited experimental budget, we examined the effects of different training data designs, i.e., screening fewer sites but more mutations on each site, or more sites but fewer mutations on each site. (**a**) Schematic visualizations of the exploration-exploitation trade-off. Given a DMS dataset, 20% of mutations of each site were withheld as test data. The experimental budget (number of variants

that can be tested) was set to 50% of the remaining variants. The allocation of the budget was controlled by an exploration ratio r, which we defined as the fraction of sites with at least one mutation being sampled in the training data. We varied the value of r = 0.5, 0.75, and 1.0 but fixed the test budget (i.e., the area of the purple region remains the same). (**b**) ECNet's performance was assessed using 37 single-mutation DMS datasets curated in the DeepSequence dataset. The box plot shows ECNet's test performance for exploration ratios 0.5, 0.75, and 1.0. The performance of unsupervised ECNet is also shown for reference. Each point represents the correlation on a single-mutation DMS dataset (n=37 datasets in total). The midline of box plots represents the median, the lower and upper hinges of the boxes correspond to the 25th and 75th percentiles, and the whiskers extend to 1.5 times the interquartile range from the hinges. The Spearman correlation increased as the exploration ratio r increased. (**c**) Pairwise performance comparison between ECNet at different exploration ratios and the unsupervised ECNet. The underlying correlation data is the same as in (**b**). Each point represents a single-mutation DMS dataset from the DeepSequence dataset. One-sided rank-sum test was used to test the statistical significance. The improvements (mean $\Delta\rho$) achieved by supervised ECNet over the unsupervised ECNet were increasing as the exploration ratio r increased.

Supplementary Table

Supplementary Table 1. Raw performance numbers on the Envision dataset. Performances of ECNet and the Envision model are evaluated using AUROC and Spearman correlation. The mean values of ten trials of five-fold cross-validation are listed.

	AUROC		Spearman correlation	
Protein	ECNet	Envision	ECNet	Envision
BRCA1 (E3 activity)	0.32	0.29	0.41	0.33
BRCA1 (BARD1 binding)	0.54	0.52	0.43	0.38
Ubiquitin (E1)	0.59	0.53	0.72	0.71
UBE4B (U-box)	0.48	0.41	0.52	0.48
PSD95 (pdz3)	0.86	0.82	0.79	0.75
Pab1	0.44	0.36	0.81	0.80
TEM-1	0.59	0.55	0.86	0.87
Ubiquitin	0.73	0.65	0.86	0.82
Yap65 (WW)	0.79	0.74	0.76	0.72
Protein G	0.88	0.83	0.91	0.87
HSP90	0.56	0.52	0.70	0.67
KKA2	0.80	0.88	0.80	0.88
