# Supplementary material for: ECNet is an evolutionary context-integrated deep learning framework for protein engineering
Source: Nat Commun. 2021 Sep 30;12:5743. doi: 10.1038/s41467-021-25976-8 (PMC8484459; doi:10.1038/s41467-021-25976-8)
Supplement: Supplementary file 3 — Description of Additional Supplementary Files [file 41467_2021_25976_MOESM3_ESM.pdf]

Description of Additional Supplementary Files

File Name: Supplementary Data 1
Description: Fitness data of TEM-1 variants obtained from experimental validation.

File Name: Supplementary Data 2
Description: List of primers used to construct TEM-1 variants.
