# Supplementary material for: ECNet is an evolutionary context-integrated deep learning framework for protein engineering
Source: Nat Commun. 2021 Sep 30;12:5743. doi: 10.1038/s41467-021-25976-8 (PMC8484459; doi:10.1038/s41467-021-25976-8)
Supplement: Supplementary file 6 — Reporting Summary [file 41467_2021_25976_MOESM6_ESM.pdf]

nature portfolio

Corresponding author(s): Huimin Zhao, Jian Peng

Last updated by author(s): Aug 27, 2021

Reporting Summary

Nature Portfolio wishes to improve the reproducibility of the work that we publish. This form provides structure for consistency and transparency in reporting. For further information on Nature Portfolio policies, see our Editorial Policies and the Editorial Policy Checklist.

Statistics

For all statistical analyses, confirm that the following items are present in the figure legend, table legend, main text, or Methods section.

n/a	Confirmed	
☐	☒	The exact sample size (*n*) for each experimental group/condition, given as a discrete number and unit of measurement
☐	☒	A statement on whether measurements were taken from distinct samples or whether the same sample was measured repeatedly
☐	☒	The statistical test(s) used AND whether they are one- or two-sided<br>*Only common tests should be described solely by name; describe more complex techniques in the Methods section.*
☒	☐	A description of all covariates tested
☐	☒	A description of any assumptions or corrections, such as tests of normality and adjustment for multiple comparisons
☐	☒	A full description of the statistical parameters including central tendency (e.g. means) or other basic estimates (e.g. regression coefficient) AND variation (e.g. standard deviation) or associated estimates of uncertainty (e.g. confidence intervals)
☐	☒	For null hypothesis testing, the test statistic (e.g. F, t, r) with confidence intervals, effect sizes, degrees of freedom and P value noted<br>*Give P values as exact values whenever suitable.*
☒	☐	For Bayesian analysis, information on the choice of priors and Markov chain Monte Carlo settings
☒	☐	For hierarchical and complex designs, identification of the appropriate level for tests and full reporting of outcomes
☒	☐	Estimates of effect sizes (e.g. Cohen's *d*, Pearson's *r*), indicating how they were calculated

Our web collection on statistics for biologists contains articles on many of the points above.

Software and code

Policy information about availability of computer code

Data collection	No software was used for data collection.
Data analysis	Our ECNet software is available at https://github.com/luoyunan/ECNet. The software was built on Python 3.7, PyTorch 1.4.0, Numpy 1.18.5, Scipy 1.4.1, Numba 0.45.1, Bio Python 1.78, SciKit-Learn 0.24.1, Pandas 1.2.3, msgpack-python 0.5.6, and TAPE 0.4 (https://github.com/songlab-cal/tape). The PacBio data of TEM-1 validation experiment was analyzed using the TADA workflow (https://github.com/h3abionet/TADA). HHblits (https://github.com/soedinglab/hh-suite) and CCMpred (https://github.com/soedinglab/CCMpred) were used to search and process homologous sequences of proteins. FoldX 5 (http://foldxsuite.crg.eu/) was used for stability analysis.

For manuscripts utilizing custom algorithms or software that are central to the research but not yet described in published literature, software must be made available to editors and reviewers. We strongly encourage code deposition in a community repository (e.g. GitHub). See the Nature Portfolio guidelines for submitting code & software for further information.

Data

Policy information about availability of data

All manuscripts must include a data availability statement. This statement should provide the following information, where applicable:
- Accession codes, unique identifiers, or web links for publicly available datasets
- A description of any restrictions on data availability
- For clinical datasets or third party data, please ensure that the statement adheres to our policy

The public datasets generated or curated in previous publications are available through the following links: Envision dataset (https://doi.org/10.1016/j.cels.2017.11.003); DeepSequence dataset (https://doi.org/10.1038/s41592-018-0138-4); Single and double mutants fitness (https://doi.org/10.1038/s41588-019-0432-9, https://doi.org/10.1016/j.jmb.2019.03.020, https://doi.org/10.1038/s41467-019-12101-z); TEM-1 single-mutation and double-mutation

mutants fitness data (https://doi.org/10.1093/molbev/msu081, https://doi.org/10.1016/j.jmb.2019.03.020); High-order avGFP fitness (https://doi.org/10.1038/nature17995); Inhibitor-resistant TEM-1 variants (downloaded from https://externalwebapps.lahey.org/studies/TEMTable.aspx and deposited at https://doi.org/10.6084/m9.figshare.16516608.v1); Homologous sequences and fitness data of viral proteins (https://doi.org/10.1126/science.abd7331); The PDB structure of TEM-1 used in this study is available on RCSB under accession code 1XPB (https://www.rcsb.org/structure/1XPB). The fitness data of TEM-1 validation experiment is available in the Supplementary Table S1.

Field-specific reporting

Please select the one below that is the best fit for your research. If you are not sure, read the appropriate sections before making your selection.

[x] Life sciences [ ] Behavioural & social sciences [ ] Ecological, evolutionary & environmental sciences

For a reference copy of the document with all sections, see nature.com/documents/nr-reporting-summary-flat.pdf

Life sciences study design

All studies must disclose on these points even when the disclosure is negative.

Sample size	No statistical methods were used to determine sample size before hand. We chose a sample size that could be easily accessed while also provide sufficient statistical power when evaluating ECNet.
Data exclusions	No data is excluded.
Replication	In ampicillin resistance assay of TEM-1 mutants, six groups of biological replicates for each of tested ampicillin concentrations were performed independently to each other within the same experimental period. Based on the fitness value calculation, no significant differences of these six biological replicates were observed which justified that all of these replication attempts were successful.
Randomization	All the mutants were created based on ECNet's predictions rather than being created randomly. All the plasmids for the predicted mutants were created and confirmed individually. Then the plasmid libraries were created by mixing equal amount of each mutant plasmid following the order of the numerically labeled mutant plasmid. There was no randomization evolved in the whole process.
Blinding	Authors were blinded to the validation set and the test set when developing and evaluating computational models.

Reporting for specific materials, systems and methods

We require information from authors about some types of materials, experimental systems and methods used in many studies. Here, indicate whether each material, system or method listed is relevant to your study. If you are not sure if a list item applies to your research, read the appropriate section before selecting a response.

Materials & experimental systems

n/a	Involved in the study
[x]	[ ] Antibodies
[x]	[ ] Eukaryotic cell lines
[x]	[ ] Palaeontology and archaeology
[x]	[ ] Animals and other organisms
[x]	[ ] Human research participants
[x]	[ ] Clinical data
[x]	[ ] Dual use research of concern

Methods

n/a	Involved in the study
[x]	[ ] ChIP-seq
[x]	[ ] Flow cytometry
[x]	[ ] MRI-based neuroimaging
